# Additivity of Ileal Amino Acid Digestibility in Diets Containing Corn, Soybean Meal, and Corn Distillers Dried Grains with Solubles for Male Broilers

**DOI:** 10.3390/ani10060933

**Published:** 2020-05-28

**Authors:** Su Hyun An, Jung Yeol Sung, Hwan-Ku Kang, Changsu Kong

**Affiliations:** 1 Department of Animal Science, Kyungpook National University, Sangju 37224, Korea; woobi89@gmail.com; 2 Monogastric Animal Feed Research Institute, Konkuk University, Seoul 05029, Korea; jungyeolsung@gmail.com; 3 Institute of Poultry Science, National Institute of Animal Science, RDA, Pyeongchang 25340, Korea; magic100@korea.kr

**Keywords:** additivity, amino acids, ileal digestibility, feed ingredients, broiler

## Abstract

**Simple Summary:**

There is limited information on the additivity on ileal digestibility of amino acids (AAs) in poultry diets containing corn distillers dried grains with solubles. The aim of this study was to test additivity of two types of ileal digestibility of AAs in mixed diets containing corn, soybean meal, and corn distillers dried grains with solubles in male Ross 308 broiler chickens. The apparent ileal digestibility of some amino acids in feed ingredients used in the study was not additive in mixed diets, whereas the standardized ileal digestibility of most amino acids was additive. Based on the results, standardized ileal digestibility values are more additive in poultry diets containing various feed ingredients compared with apparent ileal digestibility values.

**Abstract:**

The aim was to test additivity of apparent ileal digestibility (AID) and standardized ileal digestibility (SID) of amino acids (AAs) in mixed diets for Ross 308 broiler chickens. Two hundred and eighty-eight, 20-d-old male broiler chickens were assigned to one of six diets, with six birds per cage using a randomized complete block design. The diets consisted of a nitrogen-free diet, three diets containing corn, soybean meal (SBM), and corn distillers dried grains with solubles (CDDGS) as the sole source of nitrogen, respectively, and two mixed diets containing corn, SBM or CDDGS. Chromic oxide was added to the diets as an indigestible index. On day 24, birds were euthanized for ileal digesta collection. Relative proportion of the basal endogenous loss of AAs to total ileal outflow of AAs in corn was greater (*p* < 0.05) than that of SBM and CDDGS. For the corn-SBM and corn-SBM-CDDGS mixed diets, the AID of AAs differed (*p* < 0.05) from the predicted values. No difference was observed between the measured and predicted SID of AA. In conclusion, the SID of AAs is more additive in mixed diets containing corn, SBM, or CDDGS compared to AID values.

## 1. Introduction

Additivity of nutrients is fundamental for accurate diet formulation and it has been accepted that digestible amino acid (AA) values are more additive than total AA values for diet formulation because animals cannot use all the AAs in feed ingredients [1].

The AA digestibility may vary depending on various factors such as the digestive ability, age of animal, type of feed ingredient, and digestible AA contents in feed ingredients [2]. Some suggest that ileal digestibility rather than total tract digestibility of AAs is a more acceptable value for poultry diet formulation because of modification of AAs by microbes present in the hindgut [3]. Depending on the correction of basal endogenous losses (BELs) of AAs, ileal digestibility can be categorized into apparent (AID) and standardized ileal digestibility (SID). However, there have been several studies showing that AID values are less additive than SID because AID might be underestimated for ingredients containing low protein because of a greater portion of BEL of AAs in ileal digesta [1].

Using alternative feed ingredients other than corn or soybean meal (SBM) has increased because of price fluctuation of these two conventional ingredients. Demand for biofuel production with corn increased the production of corn distillers dried grains with solubles (CDDGS), which is one of common alternative ingredients used for energy and protein supply in poultry diets. Many studies have determined the ileal digestibility of AAs in various feed ingredients, including CDDGS for broilers [2,4,5,6]. However, there is a dearth of information on the additivity of digestible AAs from CDDGS in a mixed diet for broiler chickens. Therefore, the aim of the present study was to test the additivity of the AA digestibility in a mixed diet containing corn, SBM, or CDDGS for 24-day-old male broiler chickens.

## 2. Materials and Methods

Experimental procedures were approved by the Institutional Animal Care and Use Committee at Kyungpook National University, Republic of Korea (approval number: KNU2019-125).

### 2.1. Test Ingredients

Corn, SBM, and CDDGS used in this study were analyzed for AA content as shown in Table 1.

### 2.2. Dietary Treatments

The feed ingredient composition for the experimental diets is shown in Table 2. A nitrogen-free diet (NFD) was formulated to estimate the BEL of AAs. Three diets were formulated to contain either corn, SBM, or CDDGS as the sole source of AAs. Two diets containing corn and SBM, or corn, SBM, and CDDGS were formulated to test the additivity of the AID and SID of AAs in mixed diets. Energy and nutrients, except for crude protein (CP) and AAs, met or exceeded the recommended requirements for broilers [7]. All diets contained 0.5% chromic oxide (Cr_2_O_3_) as an index for the calculation of digestibility. The analyzed CP and AA concentrations in the experimental diets are shown in Table 3.

### 2.3. Animal, Management and Experimental Design

Two-hundred and eighty-eight male broiler chicks (Ross 308) were fed a standard starter diet from day 0 to 20. On day 20, birds were individually weighed and assigned to one of six treatments with eight replicates (6 birds/cage) based on body weight, in a randomized complete block design. All birds were offered *ad libitum* access to water and feed from day 0 to 24. The birds were housed in wire-floored cages in an environmentally controlled room at 32 °C on day 0; the temperature was gradually reduced to 24 °C by day 20 and further reduced to 23 °C by day 24.

### 2.4. Sample Collection

At the end of the experiment (day 24), all birds were euthanized by CO_2_ asphyxiation. Ileal digesta samples from the distal two-thirds of the ileum, which is equivalent to the portion of the small intestine from Meckel’s diverticulum to approximately 1 cm anterior of the ileocecal junction, were collected by rinsing the ileum with distilled water and collecting the contents into clean plastic containers. The digesta samples from six birds in the same cage was pooled and stored at −20 °C for further analyses.

### 2.5. Chemical Analysis

The ingredients and experimental diet samples were dried at 105 °C for 2 h (method 930.15) [8] and ground using a mill grinder (CT 293 Cyclotec, Foss Innovation Centre, Hillerød, Denmark). Freeze-dried ileal digesta samples were ground using a coffee grinder. The dried samples of the ingredients, diets, and ileal digesta were analyzed for AA content (method 982.30) [8]. Dried diets and ileal digesta samples were analyzed for chromium content [9] which was used in calculating ileal digestibility.

### 2.6. Calculation

AID, BEL, and SID of the AAs were calculated using the following equations from Kong and Adeola [10]:

AID (%) = 100 − [(Cr_diet_ × AA_digesta_)/(Cr_digesta_ × AA_diet_)] × 100 BEL (mg/kg of DMI) = (Cr_diet_/Cr_digesta_) × AA_digesta_ SID (%) = AID + [(BEL/AA_diet_) × 100] (1)

where Cr_diet_ (g/kg) and Cr_digesta_ (g/kg) are the chromium concentrations of the diet and digesta on a DM basis, respectively; AA_diet_ (mg/kg) and AA_digesta_ (mg/kg) are the AA concentrations of the diet and digesta on a DM basis, respectively. The measured and predicted AID or SID values were calculated as reported by Kong and Adeola [10] and the values were compared to test additivity of the digestibility of AA in the mixed diet.

Predicted AID or SID (%) = [(AA_corn_ × AID or SID_corn_) + (AA_SBM_ × AID or SID_SBM_) + (AA_CDDGS_ × AID or SID_CDDGS_)]/(AA_corn_ + AA_SBM_ + AA_CDDGS_) (2)

where AA_corn_, AA_SBM_, and AA_CDDGS_ are the concentrations (%) of the contributed AA in the mixed diet, respectively. The relative proportion of BEL to total ileal outflow of AAs (RBEL %) were calculated to investigate the influence of the BEL of AAs on the values of AID of the AAs using the following equations:

Total ileal outflow of AA for feed ingredient (g/kg DM) = dietary AA content (g/kg DM) × (1 − coefficient of AID of AA) RBEL (%) = BEL (g/kg DMI)/Total ileal outflow of AA (g/kg DM) × 100(3)

### 2.7. Statistical Analysis

Data were analyzed using the mixed procedure in SAS [11]. The experimental unit was a cage. The values of AID and SID for corn, SBM, and CDDGS were presented as least squares means and compared using Tukey’s Honestly Significant Difference (HSD) test. Differences in measured and predicted values for AID or SID of the AA in mixed diets were tested using a 2-sided, one-sample t-test [1]. The significance was set at an alpha-level of 0.05.

## 3. Results and Discussion

The estimated BEL of AAs in the ileal digesta are shown in Table 4. The BEL was lower in this study compared with values from previous studies [1,12,13]. The differences in BEL of AAs between the present and previous studies may be attributed, in part, to the difference in age of the birds, methods used to determine BEL including the nutrient composition of NFD, duration of feeding NFD, and types of indigestible index used for the BEL calculation [14].

Ileal digestibility rather than the total tract digestibility of AAs is commonly used because most of digestion and absorption of AAs are completed before the end of the small intestine and microbial modifications of the AAs occurs in the hindgut [3,10]. The SID of CP and AAs are considered as the more reliable value for the formulation of poultry diets in comparison with the AID because the SID values are calculated by correcting AID for BEL of CP and AAs [10]. The determined AID and SID of AAs in feed ingredients were affected differently depending on the AA compositions in this study. The AID and SID of AAs in corn, SBM, and CDDGS are shown in Table 5 and Table 6. The AID of indispensable AAs in corn, SBM, and CDDGS ranged from 74.6% (Thr) to 92.1% (Leu), from 88.5% (Thr) to 95.9% (Arg), and from 70.1% (Thr) to 89.5% (Leu), respectively. The lowest AID of Thr in corn, SBM, and CDDGS found here are in agreement with previous findings [1,12,13]. This may be attributed to a greater concentration of Thr in BEL. One of major components in BEL is the mucin protein, which is composed of a large amount of Thr [1]. The AID of AAs in corn and SBM were within the range reported in previous studies [1,15]. However, the estimated AID of AAs in CDDGS in this study was higher than those previously reported [16]. The SID of AAs for corn, SBM, and CDDGS were within the range reported in previous studies [1,15,17,18]. The SID of the indispensable AAs in corn, SBM, and CDDGS ranged from 91.4% (Thr) to 98.0% (Met), from 93.5% (Thr) to 97.4% (Met), and from 73.5% (Lys) to 89.5% (Leu), respectively. The poor SID of Lys in CDDGS is in agreement with previously reported results in broilers [12,19] and pigs [20]. The lower ileal digestibility of Lys in CDDGS is attributed to heat damage of Lys, through the Maillard reaction, during the drying process [21,22]. The Maillard reaction is a chemical reaction between reducing sugars and AAs. Lys is more susceptible to heat damage and the free amino group of Lys easily reacts with reducing sugar. The altered chemical structure of Lys can interfere with the absorption of AAs and reduce the efficiency of proteolytic enzyme activity [22].

The values for RBEL in the feed ingredients were calculated to investigate the relative influence of BEL on AID of the AAs in feed ingredients (Table 7). The value for the total ileal AA outflow for feed ingredient is calculated by multiplying dietary AA content and indigestibility of dietary AAs. The calculated value represents the sum of the indigested AAs derived from ingredients and excreted AAs from the body during the digestion process. Calculated RBEL and contents or indigestibility of the AAs are in reciprocal proportion; whereby the RBEL values increased as the contents and indigestibility of AAs decreased. In this study, corn had the greatest RBEL for all AAs, followed by SBM and CDDGS (*p* < 0.05). This can be explained by the low AA content in the corn. Whereas, the lowest RBEL for CDDGS may be because of the combined effect of the greater AA content and less digestibility of AAs in CDDGS compared with corn. In addition, SBM had a greater AID of AAs than corn, and the RBEL values for SBM were less than those for corn. This may be attributed to the relatively greater impact of AA content in SBM compared to digestibility.

Additivity of digestibility of AAs in a mixed diet is important for accurate feed formulation. In this study, the difference between the measured and the predicted digestibility of AAs in the mixed diet were used for the additivity test. If there is no difference between the measured and the predicted digestibility, digestibility of AAs in the mixed diet could be predicted by using AA digestibility of individual ingredients and their proportion in the mixed diet. For the corn-SBM mixed diet, measured values for AID in 5 of 17 AAs differed from predicted values (*p* < 0.05), however, there were no differences in the SID of AAs (Table 8). These results are in agreement with previous studies [1,15]. Kong and Adeola [10] reported that there were differences between the measured and the predicted AID of eight AAs for broilers fed a corn-SBM based diet, whereas the SID of AAs did not differ between the measured and the predicted values, except Cys. The lack of additivity for AID of AAs in the corn-SBM mixed diet may be attributed to the underestimated AID of AAs in corn. The corn had high RBEL for most of the AAs, indicating an overestimation of AAs in the total ileal outflow, which underestimates the AID of the AAs. In the corn-SBM-CDDGS mixed diet, the measured AID of two of 17 AAs differed from the predicted values, whereas the measured SID of all AAs in the mixed diet were not different from the predicted values, except Arg (*p* < 0.05, Table 9). These results are in agreement with a previous study [12]. The corn-SBM-CDDGS mixed diet had fewer differences between the measured and the predicted AID of AAs than the corn-SBM mixed diet. This may be attributed to a reduction of corn in the mixed diet and an increase in DDGS which has relatively less RBEL compared with the corn.

## 4. Conclusions

The present study shows that the AID of AAs could be underestimated when the digestibility is determined for low-CP ingredients such as corn, which has a relatively greater RBEL compared with other ingredients, which have lower RBEL and high CP. In addition, the use of the AID of AAs in corn for predicting digestibility of AAs in the mixed diet containing corn cause an underestimation of AA digestibility; whereas the additivity was improved with ingredients having relatively greater CP and lower RBEL compared to the corn. The present study also suggests that the SID of AAs in individual ingredient is more accurate in predicting the digestibility of AAs in a mixed diet compared to the AID. In conclusion, the SID of AAs is more additive in mixed diets containing corn, SBM, or CDDGS compared to AID values.

## Figures and Tables

**Table 1:** Analyzed amino acid composition of feed ingredients (%, as-fed basis).

Item	Feed Ingredient		
	Corn	SBM	CDDGS
Indispensable amino acid			
Arg	0.35	3.15	1.12
His	0.21	1.09	0.65
Ile	0.26	2.03	0.95
Leu	0.90	3.48	3.02
Lys	0.24	2.75	0.84
Met	0.16	0.52	0.55
Phe	0.40	2.30	1.25
Thr	0.29	1.83	1.02
Val	0.38	2.05	1.26
Dispensable amino acid			
Ala	0.55	2.04	1.94
Asp	0.51	5.03	1.72
Cys	0.16	0.63	0.54
Glu	1.44	8.00	4.13
Gly	0.28	1.88	1.04
Pro	0.71	2.41	2.20
Ser	0.37	2.18	1.24
Tyr	0.17	1.34	0.76

SBM: soybean meal; CDDGS: corn distillers dried grains with solubles.

**Table 2:** Ingredient composition (%) of the experimental diets (as-fed basis).

Item	Dietary Treatments					
	NFD	Corn	SBM	CDDGS	Corn-SBM	Corn-SBM-CDDGS
Ingredient %						
Corn	-	92.72	-	-	56.90	46.91
SBM	-	-	41.84	-	32.76	22.90
CDDGS	-	-	-	73.00	-	20.00
Cornstarch	29.38	2.00	49.29	18.73	2.00	2.00
Sucrose	55.00	-	-	-	-	-
Soybean oil	4.00	-	4.00	4.00	3.50	3.50
Dicalcium phosphate	2.14	1.78	1.70	0.79	1.59	1.38
Limestone	1.88	2.00	1.76	2.14	1.85	1.93
Vitamin premix ^1^	0.20	0.20	0.20	0.20	0.20	0.20
Mineral premix ^2^	0.20	0.20	0.20	0.20	0.20	0.20
Salt	-	0.40	0.40	0.40	0.40	0.40
Chromic oxide	0.50	0.50	0.50	0.50	0.50	0.50
Choline chloride	0.26	0.20	0.11	0.04	0.10	0.08
Cellulose	5.00	-	-	-	-	-
Sodium bicarbonate	0.75	-	-	-	-	-
Potassium chloride	0.30	-	-	-	-	-
Magnesium oxide	0.09	-	-	-	-	-
Potassium carbonate	0.30	-	-	-	-	-
Total	100.00	100.00	100.00	100.00	100.00	100.00
Calculated nutrient and energy						
AME, kcal/kg	3384	3188	3362	2908	3017	3001
Crude protein, %	0.0	7.1	20.0	20.0	20.0	20.0
Calcium, %	1.00	1.00	1.00	1.00	1.00	1.00
Non-phytate, P %	0.45	0.45	0.45	0.45	0.45	0.45

NFD: nitrogen-free diet; SBM: soybean meal; CDDGS: corn distillers dried grains with solubles; AME: apparent metabolizable energy; P: phosphorus. ^1^ Supplies the following per kilogram of diet: vitamin A, 24,000 IU; vitamin D_3_, 8000 IU; vitamin E, 160 mg/kg; vitamin K_3_, 8 mg/kg; vitamin B_1_, 8 mg/kg; vitamin B_2_, 20 mg/kg; vitamin B_6_, 12 mg/kg; pantothenic acid, 40 mg/kg; folic acid, 4 mg/kg; niacin, 12 mg/kg; ^2^ Supplies the following per kilogram of diet: Fe, 120 mg/kg; Cu, 320 mg/kg; Zn, 200 mg/kg; Mn, 240 mg/kg; Co, 2 mg/kg; Se, 0.6 mg/kg; I, 2.5 mg/kg.

**Table 3:** Analyzed amino acids composition of the experimental diets (%, as-fed basis).

Item	Dietary Treatment					
	NFD	Corn	SBM	CDDGS	Corn-SBM	Corn-SBM-CDDGS
Indispensable amino acid						
Arg	-	0.35	1.58	0.81	1.38	1.12
His	-	0.21	0.55	0.47	0.53	0.50
Ile	-	0.25	1.04	0.68	0.91	0.80
Leu	-	0.85	1.75	2.17	1.80	1.93
Lys	-	0.23	1.39	0.62	1.19	0.94
Met	-	0.13	0.26	0.33	0.29	0.30
Phe	-	0.35	1.17	0.92	1.07	1.01
Thr	-	0.28	0.93	0.74	0.87	0.81
Val	0.02	0.36	1.07	0.91	0.99	0.95
Dispensable amino acid						
Ala	-	0.53	1.02	1.40	1.08	1.17
Asp	0.01	0.49	2.54	1.26	2.23	1.83
Cys	-	0.18	0.29	0.40	0.32	0.37
Glu	0.02	1.35	4.11	3.09	3.86	3.57
Gly	0.01	0.29	0.94	0.76	0.87	0.80
Pro	-	0.67	1.18	1.56	1.28	1.33
Ser	-	0.34	1.11	0.90	1.05	0.98
Tyr	-	0.15	0.56	0.55	0.51	0.51

NFD: Nitrogen-free diet; SBM: Soybean meal; CDDGS: corn distillers dried grains with solubles.

**Table 4:** Ileal basal endogenous losses of amino acids for broiler chickens fed the nitrogen-free diet (mg/kg DMI) ^1^.

Item	Basal Endogenous Losses of Amino Acid
Indispensable amino acid	
Arg	156
His	69
Ile	208
Leu	312
Lys	156
Met	87
Phe	278
Thr	469
Val	260
Dispensable amino acid	
Ala	139
Asp	469
Cys	191
Glu	677
Gly	226
Pro	278
Ser	434
Tyr	122

DMI: dry matter intake. ^1^ Values were determined using the pooled samples from eight replicate cages of six birds per cage.

**Table 5:** Apparent ileal amino acid digestibility (%) of broilers fed corn-, SBM-, and CDDGS-based diets.

Item	Apparent Ileal Digestibility			SEM	*p*-Value
	Corn	SBM	CDDGS		
Indispensable amino acid					
Arg	90.2 ^b^	95.9 ^a^	85.8 ^c^	0.55	<0.001
His	88.4 ^b^	93.4 ^a^	80.5 ^c^	0.75	<0.001
Ile	86.4 ^b^	92.7 ^a^	80.3 ^c^	0.78	<0.001
Leu	92.1 ^a^	92.5 ^a^	88.1 ^b^	0.47	<0.001
Lys	82.1 ^b^	93.4 ^a^	70.9 ^c^	1.14	<0.001
Met	91.3 ^a^	94.1 ^a^	86.0 ^b^	0.88	<0.001
Phe	88.7 ^b^	92.2 ^a^	82.0 ^c^	0.89	<0.001
Thr	74.6 ^b^	88.5 ^a^	70.1 ^b^	1.47	<0.001
Val	86.0 ^b^	91.8 ^a^	79.5 ^c^	0.87	<0.001
Dispensable amino acid					
Ala	91.1 ^b^	93.1 ^a^	86.6 ^c^	0.47	<0.001
Asp	83.2 ^b^	90.5 ^a^	70.8 ^c^	1.21	<0.001
Cys	85.0 ^a^	86.8 ^a^	77.6 ^b^	1.26	<0.001
Glu	91.6 ^a^	94.2 ^a^	86.4 ^b^	0.69	<0.001
Gly	79.7 ^b^	89.2 ^a^	71.3 ^c^	1.09	<0.001
Pro	88.5 ^b^	91.9 ^a^	85.0 ^c^	0.46	<0.001
Ser	82.3 ^b^	90.3 ^a^	77.2 ^c^	1.24	<0.001
Tyr	85.6 ^c^	93.7 ^a^	87.8 ^b^	0.52	<0.001

^a–c^ Least squares means within a row without a common superscript differ (*p* < 0.05). SBM: soybean meal; CDDGS: corn distillers dried grains with solubles; SEM: standard error of the mean.

**Table 6:** Standardized ileal amino acid digestibility (%) of broilers fed corn-, SBM-, and CDDGS-based diets.

Item	Standardized Ileal Digestibility			SEM	*p*-Value
	Corn	SBM	CDDGS		
Indispensable amino acid					
Arg	94.7 ^a^	96.9 ^a^	87.7 ^b^	0.55	<0.001
His	91.7 ^a^	94.7 ^a^	82.0 ^b^	0.75	<0.001
Ile	94.7 ^a^	94.7 ^a^	83.4 ^b^	0.78	<0.001
Leu	95.7 ^a^	94.3 ^a^	89.5 ^b^	0.47	<0.001
Lys	88.9 ^b^	94.5 ^a^	73.5 ^c^	1.14	<0.001
Met	98.0 ^a^	97.4 ^a^	88.6 ^b^	0.88	<0.001
Phe	96.6 ^a^	94.6 ^a^	85.1 ^b^	0.89	<0.001
Thr	91.4 ^a^	93.5 ^a^	76.5 ^b^	1.47	<0.001
Val	93.2 ^a^	94.2 ^a^	82.4 ^b^	0.87	<0.001
Dispensable amino acid					
Ala	93.8 ^a^	94.4 ^a^	87.5 ^b^	0.47	<0.001
Asp	92.7 ^a^	92.3 ^a^	74.5 ^b^	1.21	<0.001
Cys	95.6 ^a^	93.4 ^a^	82.3 ^b^	1.26	<0.001
Glu	96.7 ^a^	95.8 ^a^	88.6 ^b^	0.69	<0.001
Gly	87.5 ^a^	91.6 ^a^	74.3 ^b^	1.09	<0.001
Pro	92.6 ^a^	94.2 ^a^	86.8 ^b^	0.46	<0.001
Ser	95.1 ^a^	94.2 ^a^	82.0 ^b^	1.24	<0.001
Tyr	93.7 ^b^	95.9 ^a^	90.0 ^c^	0.52	<0.001

^a–c^ Least squares means within a row without a common superscript differ (*p* < 0.05). SBM: soybean meal; CDDGS: corn distillers dried grains with solubles; SEM: standard error of the mean.

**Table 7:** Relative proportion of BEL of AAs to total ileal outflow of AAs (%) in broilers fed corn-, SBM-, or CDDGS -based diets.

Item	Relative Proportion of BEL of AAs to Total Ileal Outflow of AAs			SEM	*p*-Value
	Corn	SBM	CDDGS		
Indispensable amino acid					
Arg	42.9 ^a^	23.2 ^b^	12.5 ^c^	1.43	<0.001
His	26.7 ^a^	18.5 ^b^	7.0 ^c^	0.80	<0.001
Ile	57.2 ^a^	26.4 ^b^	14.3 ^c^	1.52	<0.001
Leu	43.4 ^a^	22.9 ^b^	11.1 ^c^	1.02	<0.001
Lys	35.5 ^a^	16.4 ^b^	8.0 ^c^	0.95	<0.001
Met	72.0 ^a^	55.1 ^b^	17.6 ^c^	3.11	<0.001
Phe	65.7 ^a^	29.7 ^b^	15.6 ^c^	2.05	<0.001
Thr	61.9 ^a^	42.4 ^b^	19.6 ^c^	2.32	<0.001
Val	48.2 ^a^	28.8 ^b^	12.9 ^c^	1.66	<0.001
Dispensable amino acid					
Ala	27.7 ^a^	18.9 ^b^	6.8 ^c^	0.82	<0.001
Asp	53.4 ^a^	18.8 ^b^	11.7 ^c^	1.60	<0.001
Cys	66.1 ^a^	49.2 ^b^	19.7 ^c^	3.31	<0.001
Glu	56.1 ^a^	27.5 ^b^	14.9 ^c^	1.63	<0.001
Gly	35.9 ^a^	21.5 ^b^	9.5 ^c^	0.98	<0.001
Pro	33.6 ^a^	27.9 ^b^	10.9 ^c^	1.01	<0.001
Ser	67.9 ^a^	39.0 ^b^	19.7 ^c^	2.62	<0.001
Tyr	52.8 ^a^	33.3 ^b^	16.7 ^c^	1.79	<0.001

^a–c^ Least squares means within a row without a common superscript differ (*p* < 0.05). BEL: basal endogenous loss; AAs: Amino Acids; SBM: soybean meal; CDDGS: corn distillers dried grains with solubles; SEM: standard error of the mean.

**Table 8:** Measured and predicted values for AID and SID (%) of AAs and the difference between the measured and the predicted AID and SID of AAs in male broilers fed a corn and SBM mixed diet.

Item	AID					SID				
	Measured	Predicted	Difference ^1^	SE	*p*-Value	Measured	Predicted	Difference	SE	*p*-Value
Indispensable amino acid										
Arg	95.3	95.0	0.3	0.29	0.495	96.4	96.5	−0.1	0.29	0.797
His	93.1	92.1	1.0	0.39	0.132	94.4	93.9	0.5	0.39	0.381
Ile	92.6	91.5	1.1	0.45	0.134	94.8	94.7	0.3	0.45	0.724
Leu	93.0	92.3	0.6	0.45	0.399	94.7	94.7	0.0	0.45	0.983
Lys	93.3	91.9	1.4	0.34	0.057	94.6	93.8	0.9	0.34	0.125
Met	94.6	93.1	1.5	0.30	0.013	97.6	97.6	0.0	0.30	0.970
Phe	92.7	91.4	1.3	0.48	0.134	95.3	95.1	0.3	0.48	0.734
Thr	89.1	85.5	3.7	0.65	0.027	94.5	93.1	1.5	0.65	0.159
Val	92.1	90.3	1.8	0.51	0.048	94.8	93.9	0.9	0.51	0.300
Dispensable amino acid										
Ala	92.9	92.4	0.5	0.41	0.441	94.2	94.2	0.0	0.41	1.000
Asp	90.9	89.4	1.5	0.53	0.086	93.0	92.4	0.7	0.53	0.426
Cys	88.5	86.2	2.2	0.81	0.101	94.4	94.1	0.4	0.81	0.765
Glu	94.2	93.6	0.6	0.35	0.264	95.9	96.0	−0.1	0.35	0.869
Gly	89.2	87.3	1.9	0.55	0.048	91.8	90.8	1.1	0.55	0.237
Pro	91.9	90.7	1.2	0.82	0.352	94.1	93.7	0.4	0.82	0.755
Ser	91.1	88.4	2.7	0.60	0.020	95.2	94.4	0.9	0.60	0.348
Tyr	93.3	92.2	1.0	0.40	0.117	95.6	95.5	0.2	0.40	0.761

AID: apparent ileal digestibility; SID: standardized ileal digestibility; AAs: Amino Acids; SBM: soybean meal; SE: standard error. ^1^ Calculated as measured–predicted. Data represents mean from four replicates per treatment.

**Table 9:** Measured and predicted values for the AID and SID (%) of AAs and difference between measured and predicted AID and SID of AAs in male broilers fed a corn, SBM, and CCDGS mixed diet.

Item	AID					SID				
	Measured	Predicted	Difference ^1^	SE	*p*-Value	Measured	Predicted	Difference	SE	*p*-Value
Indispensable amino acid										
Arg	92.1	93.0	−0.9	0.31	0.086	93.3	94.5	−1.2	0.31	0.037
His	89.5	88.9	0.7	0.37	0.288	90.2	89.9	0.3	0.37	0.497
Ile	88.9	88.6	0.2	0.41	0.723	91.1	91.5	−0.4	0.41	0.462
Leu	91.3	90.9	0.4	0.31	0.420	92.5	92.6	−0.1	0.31	0.790
Lys	87.9	87.8	0.1	0.45	0.861	89.3	89.6	−0.3	0.45	0.635
Met	91.9	90.5	1.5	0.45	0.071	94.2	94.2	0.5	0.45	0.453
Phe	89.8	88.9	0.9	0.50	0.239	91.9	91.9	0.1	0.50	0.952
Thr	83.8	81.1	2.7	0.71	0.037	88.5	87.5	1.0	0.71	0.311
Val	88.4	87.2	1.2	0.56	0.167	90.6	90.1	0.5	0.56	0.546
Dispensable amino acid										
Ala	90.2	90.3	−0.1	0.33	0.755	91.4	91.9	−0.5	0.33	0.344
Asp	85.5	85.6	−0.1	0.61	0.891	87.5	88.4	−0.8	0.61	0.350
Cys	86.5	83.3	3.2	0.77	0.026	90.6	90.6	1.5	0.77	0.190
Glu	91.5	91.7	−0.3	0.36	0.592	92.8	92.8	−0.8	0.36	0.103
Gly	83.4	82.8	0.6	0.61	0.503	86.2	86.2	0.0	0.61	0.966
Pro	89.4	88.7	0.7	0.26	0.113	91.5	91.5	0.2	0.26	0.683
Ser	87.1	85.2	1.9	0.63	0.081	90.5	90.5	0.5	0.63	0.568
Tyr	91.4	90.8	0.6	0.32	0.255	93.6	93.8	−0.1	0.32	0.800

AID: apparent ileal digestibility; SID: standardized ileal digestibility; AAs: Amino Acids; SBM: soybean meal; CDDGS: corn distillers dried grains with solubles; SE: standard error. ^1^ Calculated as measured–predicted. Data represents mean from four replicates per treatment.
